# Clinical Features and Prognosis in Chinese Patients With Dipeptidyl–Peptidase–Like Protein 6 Antibody–Associated Encephalitis

**DOI:** 10.3389/fneur.2021.817896

**Published:** 2022-01-14

**Authors:** Ailiang Miao, Yongwei Shi, Xiaoshan Wang, Jianqing Ge, Chuanyong Yu

**Affiliations:** ^1^Department of Neurology, Affiliated Brain Hospital of Nanjing Medical University, Nanjing, China; ^2^Department of Video-Electroencephalogram, Affiliated Brain Hospital of Nanjing Medical University, Nanjing, China; ^3^Department of Neurology, Taizhou Fourth People's Hospital, Taizhou, China

**Keywords:** encephalitis, dipeptidyl–peptidase–like protein 6, electroclinical symptoms, migrating myoclonus, prognosis

## Abstract

**Objectives:**

Anti-dipeptidyl–peptidase–like protein 6 (anti-DPPX) encephalitis an extremely rare type of immune-mediated encephalitis. This study aimed to analyze the electroclinical characteristics and prognosis of anti-DPPX encephalitis.

**Methods:**

Five patients (all male) with anti-DPPX encephalitis in East China from January 2016 to October 2021 was retrospective analyzed. Electroclinical features and outcomes were reviewed.

**Results:**

All five patients were male. The media age at disease onset was 32 years old with a range of 14–56 years. The main symptoms included psychiatric disturbances (2/5), amnesia (4/5), confusion (3/5), and seizures (3/5). Migrating myoclonus were identified in patient 4 with positive DPPX and contactin-associated protein-like 2 antibodies in blood. All of the patients had positive DPPX antibodies in serum. Only one of them had positive antibody in the cerebrospinal fluid. EEG showed diffuse slowing in two patients, but no epileptiform discharges were observed. Eighty percent (4/5) of the patients showed normal brain magnetic resonance imaging. After immunotherapy, improvement of neuropsychiatric symptoms from all of the patients was observed. Over a mean follow-up of 30.8 weeks, all of the patients had marked improvement in the modified Rankin Scale. To date, no tumors were not observed in any patients.

**Conclusions:**

Anti-DPPX encephalitis mainly presents as neuropsychiatric symptoms. Cooperation of DPPX antibodies and CASPR2 antibodies might have contributed to the migration of myoclonus in the patient 4. Prompt immunotherapy often results in improvement.

## Introduction

In 2013, anti-dipeptidyl–peptidase–like protein 6 (anti-DPPX) encephalitis was first reported (1). The DPPX in the neuronal dendrites and soma is involved in attenuation of back-propagation of action potentials and somatodendritic signal integration, which constitute a regulatory subunit of Kv4.2 potassium channel (2, 3). The clinical symptoms included brain disorders (amnesia, delirium, psychosis, depression, seizures), brainstem disorders (eye movement disturbances, ataxia, dysphagia, dysarthria, respiratory failure), sleep disturbance, central hyperexcitability (myoclonus, diffuse rigidity, exaggerated startle response, hyperreflexia), and dysautonomia (gastrointestinal tract issues, gastroparesis, and constipation, bladder issues, cardiac conduction system problems, thermoregulation issues) (4).

The manifestations of Chinese patients were not reported. Here, the clinical features of the 5 patients with anti-DPPX encephalitis were reviewed, which could be conductive to correct diagnosis and prompt treatment.

## Methods

A total of five patients with anti-DPPX encephalitis were included from the Department of Neurology of Nanjing Brain Hospital between January 2016 and October 2021. The anti-DPPX encephalitis diagnostic criteria were as follows: (1) acute, subacute and insidious onset; (2) patients with one or more symptoms, including brain disorders (amnesia, delirium, psychosis, depression, seizures), brainstem disorders (eye movement disturbances, ataxia, dysphagia, dysarthria, respiratory failure), sleep disturbance, central hyperexcitability (myoclonus, diffuse rigidity, exaggerated startle response, hyperreflexia), and dysautonomia (gastrointestinal tract issues, gastroparesis, and constipation, bladder issues, cardiac conduction system problems, thermoregulation issues); and (3) patients with positive DPPX antibodies in serum with or without positive in cerebrospinal fluid (CSF). If patents with negative anti-DPPX antibodies in CSF, twice anti-DPPX antibodies in serum should be positive. If patients are diagnosed with other diseases, such as brain tumor, viral encephalitis, metabolic diseases, and drug poisoning, they were excluded (5).

### Patient Data

The following patient data were collected: age; sex; neurological symptoms; anti-DPPX titers; laboratory studies, video electroencephalography (VEEG), and magnetic resonance imaging (MRI); and lumbar puncture; treatment and response to treatment, and prognosis. This study was approved by the Research Ethics Committee of the Affiliated Brain Hospital of Nanjing Medical University. Clinical data was collected by the patients' medical records of hospitalization and outpatient visits. Prognosis was gathered during regular outpatient visits or telephone interviews.

Serum tumor markers, chest computed tomography (CT), thymus CT, abdominal CT, thymus ultrasonography and abdominal ultrasonography were used for screening tumor.

### Anti-neuronal Antibodies

Antibodies against cell-surface antigens and intracellular antigens were screened, including DPPX, N-methyl-D-aspartate receptor, contactin-associated protein-like 2 (CASPR2), alpha-amino-3-hydroxy-5-methyl-4-isoxazolepropionic acid receptor, leucine-rich glioma inactivated protein 1, γ-aminobutyric acid-B receptor (GABAB-R), CV2/collapsin response mediator protein 5, paraneoplastic Ma family 2, glutamic acid decarboxylase 65-kilodalton isoform (GAD65), Hu, Yo, Ri, and amphiphysin. All antibodies in CSF and serum were measured using indirect immunofluorescence technique by cell-based assays (CBA) and immunoblotting technique (Euroimmun kits, Germany).

## Results

### Clinical Presentations

We retrospectively identified five patients with anti-DPPX encephalitis (all males). Clinical features are listed in detail in Table 1. The media age at disease onset was 32 years old with a range of 14–56 years. Patient 3 was misdiagnosed with viral encephalitis for 4 years. Four patients with acute development were observed. The neurologic symptoms included psychiatric disturbances (2/5), amnesia (4/5), confusion (3/5), seizure (3/5), consciousness disturbance (1/5), ataxia (1/5), and sleep disorder (1/5).

**Table 1:** Clinical presentation in patients with anti-DPPX encephalitis.

**Patient** **number**	**Age (years)/ sex**	**Onset to diagnosis**	**Onset** **mode**	**Initial symptoms**	**Other symptoms**	**MRI**	**EEG**	**CSF**	**DPPX antibodies titer**	**Other antibody**	**Treatment**	**Tumor**	**mRS initial**	**mRS at last follow-up**	**Follow-up (months)**
1	32/M	10 days	Acute	Seizure	Amnesia	Normal	Severe diffuse polymorphic slowing	WBC 32, Normal protein	CSF (-), blood 1:100 (twice)	(-)	Immunoglobulin; Methylprednisolone; Antiepileptic drug	(-)	3	1	20
2	14/M	20 days	Acute	Psychiatric disturbances	Amnesia; Confusion	Normal	Normal	Normal WBC, protein 63 mg/dL	CSF (-), blood 1:100 (twice)	Anti-TPO >600IU/ml; Anti-TG 345.03 IU/ml	Immunoglobulin; Methylprednisolone;	(-)	3	2	26
3	33/M	4 years; Diagnosed with viral encephalitis for 4 years	Acute	Psychiatric disturbances	Fever Amnesia; Confusion; Seizure; Headache; Consciousness disturbance	Normal	Normal; Mild diffuse polymorphic slowing (after three years); Mild diffuse polymorphic slowing (after four years)	WBC 78, Normal Protein; WBC 20, Normal protein (after three years); WBC 19; protein 71.2 mg/dL (after four years)	CSF 1:1, blood 1:10	Anti-TPO126.3IU/ml; Anti-TG 63.2 IU/ml	Immunoglobulin; Methylprednisolone	(-)	5	2	70
4	17/M	10 days	Acute	Headache	Migrating myoclonus (video)	Normal	Normal	Normal WBC and protein	CSF (-); blood 1:100 (twice)	Anti-CASPR2 1:10 (twice)	Immunoglobulin (twice); Methylprednisolone; Clonazepam Valproic acid	(-)	2	1	2
5	56/M	7 months	Subacute	Amnesia	Ataxia; Seizure; Confusion; Sleep disorder	Increased signal in bilateral hippocampus	Normal	Normal WBC; protein 59 mg/dL	CSF (-), blood 1:32; CSF (-), blood 1:32 (recurrence, after 26 months)	(-)	Methylprednisolone (twice times); Mycophenolate mofetil; Antiepileptic drug	(-)	4	2	32

*Anti-TPO, antibody against thyroid peroxidase; Anti-TG, antibody against thyroglobulin; Anti-CASPR2, contactin-associated protein-like 2*.

**Figure 1:**
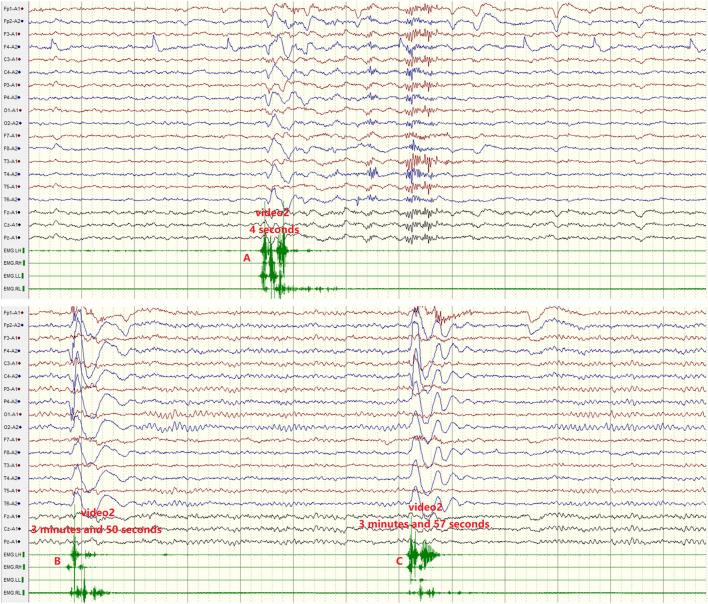
Migrating myoclonus in patient 4. **(A)** According to myoelectric activity on electromyography (EMG), EMG showed myoelectric activities of the left limbs preceding the right limbs. Electrographical artifacts were observed, along with myoclonus. **(B)** Myoclonus spreads from the right upper limb to other limbs. **(C)** Myoclonus involved the bilateral upper limbs synchronously.

We also identified migrating myoclonus in patient 4 ( Supplementary Videos 1, 2). Myoclonus in the patient varied, and could involve only one limb, or multiple limbs synchronously, or could spread from one limb to other limbs (Supplementary Videos 1, 2; Figure 1). Electrographical artifacts and myoelectric activities were observed along with myoclonus in Figure 1 (Supplementary Video 2; Figure 1).

### Physical and Laboratory Findings

**Figure 2:**
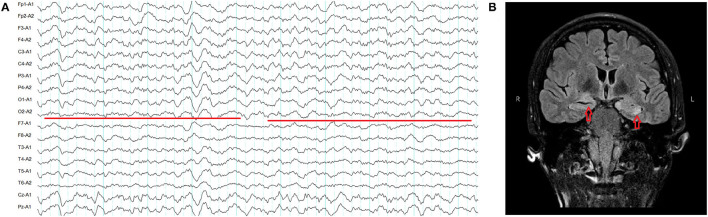
**(A)** Severe diffuse polymorphic slowing in patient 1 (red line). **(B)** Increased signal in the bilateral hippocampus in patient 5 (red arrow).

Four patients had normal MRI. EEG showed diffuse slowing in two patients with normal MRI (Table 1, Figure 2), but no epileptiform discharges were observed. An increased signal in bilateral hippocampus was observed in patient 5 with normal EEG (Figure 2).

**Figure 3:**
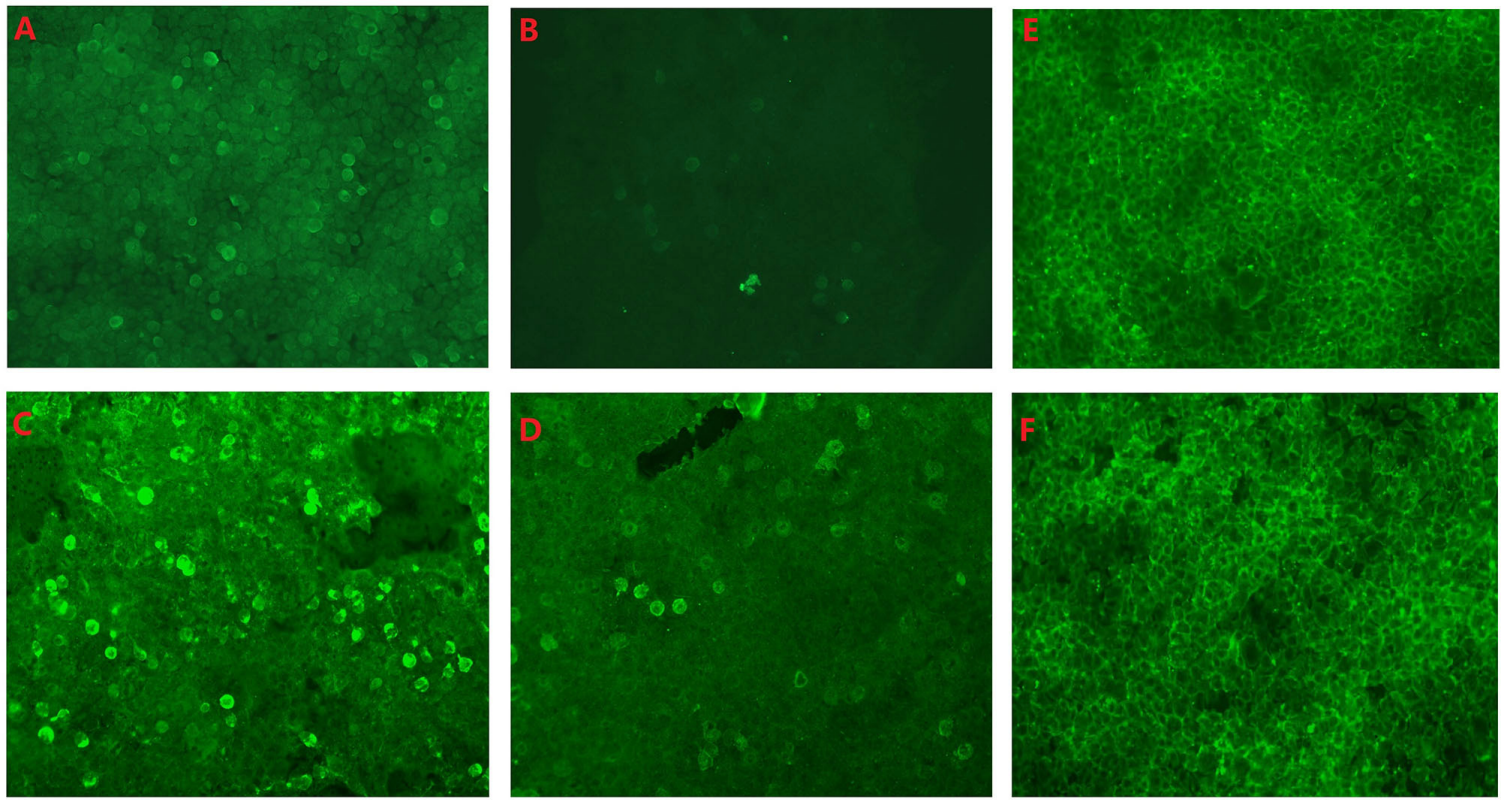
**(A)** Anti-dipeptidyl–peptidase–like protein 6 (anti-DPPX) antibody titers in serum in fifixed HEK293 cells from patient 3: 1:10. **(B)** Anti-DPPX antibody titers in cerebrospinal fluid (CSF) in fifixed HEK293 cells from patient 3: 1:1. **(C)** Anti-DPPX antibody titers in serum in fifixed HEK293 cells from patient 4: 1:100. **(D)** Anti-contactin-associated protein-like 2 antibody titers in serum in fifixed HEK293 cells from patient 4: 1:10. No antibody in CSF **(E)** and serum **(F)** was observed in fifixed HEK293 cells form the control slide. All antibodies in CSF and serum were measured using indirect immunofluorescence technique (Euroimmun kits, Germany).

All of the patients underwent lumbar puncture. Three (3/5) had elevated CSF protein ranging from 59 to 71.2 mg/dL. Two (2/5) had elevated leukocytes ranging from 19 to 78/mm^3^. All of the patients had positive DPPX antibodies in serum ranging from 1:10 to 1:100, and one of them had 1:1 antibodies titer in the CSF (Figure 3). Patient 4 had positive DPPX and CASPR2 antibodies in blood samples (Figures 3C,D). Two patients were positive for antibodies against thyroid peroxidase and thyroglobulin. The onconeuronal antibodies in all patients were negative. Virus/bacteria in CSF and oligoclonal bands in the five patients were not screened. However, toxoplasma gondii IgM, rubella virus IgM, cytomegalovirus IgM, herpes simplex virus-1 IgM, and herpes simplex virus-2 IgM in serum from four patients were negative. No tumors were found in any of the patients.

### Treatment and Prognosis

All of the patients were treated with first-line immunotherapy, including methylprednisolone and immunoglobulin (IVIG). After IVIG, methylprednisolone, clonazepam and valproic acid treatment, patient 4 still suffered from myoclonus and received another round of IVIG treatment (Table 1). Patient 5 received only methylprednisolone and did not accept IVIG. After recurrence, patients received methylprednisolone and mycophenolate mofetil treatment. During the follow-up period (range: 2 to 70 months), all of the patients showed improvement of in mRS. To date, no tumors have been observed in any patients (Table 1).

## Discussion

We report 5 new patients with anti-DPPX encephalitis in our hospital and summarize their clinical characteristics. The patients were all men with a median onset age of 32 years old. The onset pattern of the disease varied from acute to subacute. Positive DPPX antibodies in serum were observed in all of the patients, and only one of them had positive antibody in the CSF. One patient (1/5) had positive DPPX and CASPR2 antibodies in blood samples. Abnormal MRI was only observed in one patient. Three patients had normal EEG.

Kv4.2 channels are widespread in the neuronal dendrites and soma; thus, prominent clinical manifestations of DPPX autoimmunity involve cortices, cerebellar, brainstem, myelitis, and autonomic nerve (4, 6, 7). The neurologic symptoms in this study included psychiatric disturbances (2/5), amnesia (4/5), confusion (3/5), seizure (3/5), consciousness disturbance (1/5), ataxia (1/5), and sleep disorder (1/5). In a previous study, weight loss was one of the first symptoms of the disease and was not subtle (median 20 kg in the current patients) (6). The abundant level of DPPX in the myenteric plexus might explain the weight loss (1). The autonomic symptoms included diarrhea, gastroparesis, constipation, urinary symptoms, fluctuating hypothermia/hyperthermia, asymptomatic ventricular tachycardia, and cardiac arrest (4). In this study, weight loss and autonomic symptoms were not observed. The presence of gastrointestinal problems and autonomic symptoms might be dependent on ethnicity, which also might contribute to the presence of an ovarian teratoma in patients with anti-NMDAR encephalitis (8).

Symptoms of central hyperexcitability including exaggerated startle, myoclonus, diffuse rigidity, and hyperreflexia, are common in anti-DPPX encephalitis (4, 6). Myoclonus and stiffness/rigidity could be considered progressive encephalomyelitis, rigidity, and myoclonus (PERM), a syndrome predominantly described as a polioencephalo myelitis predominantly (9–12). PERM is mediated by immune responses against proteins of the GABAergic synapses, such as the glycine receptor and, less frequently, amphiphysin or GAD 65 (13, 14). Previous reports described five patients with DPPX antibodies who developed a clinical syndrome of PERM (6, 15). We did not find PERM in this study. However, we observed ictal episodes in patient 4 with positive DPPX and CASPR2 antibodies in blood. The features of these episodes: (1) were sudden and brief (Supplementary Videos 1, 2; Figure 1); (2) varied, could involve only one limb, or multiple limbs synchronously, or could spread from one limb to other limbs. No epileptic discharges were not observed during these episodes (Supplementary Videos 1, 2; Figure 1). A previous report showed myoclonus in 40% (8/20) of patients with anti-DPPX encephalitis (4). Anti-CASPR2 as membrane protein is expressed in the nervous system. It is essential for proper localization of voltage-gated potassium channels (VGKCs). Although movement disorders are rarely seen in patients with CASPR2-encephalitis, myoclonus were also observed in association with CASPR2 antibodies (16, 17). For example, myoclonic contraction of the lower limbs while standing or walking, segmental spinal myoclonus leading to trunk flexion, and dystonic posturing in both upper and lower limbs with superimposed myoclonic jerks predominantly in the left upper and lower limbs can be observed in patients with CASPR2 antibodies (18–21). However, migrating myoclonus was not reported in these patients with anti-DPPX encephalitis and anti-CASPR2 encephalitis (4, 16–21). Cooperation of DPPX antibodies and CASPR2 antibodies might have contributed to the migration of myoclonus in the patient 4. Patient 4 was treated with immunoglobulin, methylprednisolone, clonazepam, and valproic acid. After one month, the patient still suffered from myoclonus, received another round of IVIG treatment, and was discharged after improvement (Table 1).

A limitation of this study is that it enrolled only five patients. The neurologic manifestations of anti-DPPX encephalitis are diverse. Common manifestations include psychiatric disturbances, amnesia, confusion, and seizures. Migrating myoclonus could be observed in patients with anti-DPPX and anti-CARPR2 encephalitis. Patients appear to respond well to early-initiated immunotherapy.

## Data Availability Statement

The original contributions presented in the study are included in the article/Supplementary Material, further inquiries can be directed to the corresponding author.

## Ethics Statement

The studies involving human participants were reviewed and approved by the Research Ethics Committee of the Affiliated Brain Hospital of Nanjing Medical University. The patients/participants provided their written informed consent to participate in this study. Written informed consent to participate in this study was provided by the participants' legal guardian/next of kin.

## Author Contributions

AM and YS: drafting/revising the manuscript. XW: design and study supervision. JG and CY: clinical work. All authors contributed to the article and approved the submitted version.

## Funding

The work was supported by the Young Scientists Fund of the National Natural Science Foundation of China (No. 81501126), Science and Development Foundation of Nanjing Medical University (No. 2014NJMU050), Young Medical Key Talents Foundation of Jiangsu Province (No. QNRC2016053), Training Project for Young Talents of Nanjing Brain Hospital, and General project of Nanjing Municipal Health Commission (No.YKK21110).

## Conflict of Interest

The authors declare that the research was conducted in the absence of any commercial or financial relationships that could be construed as a potential conflict of interest.

## Publisher's Note

All claims expressed in this article are solely those of the authors and do not necessarily represent those of their affiliated organizations, or those of the publisher, the editors and the reviewers. Any product that may be evaluated in this article, or claim that may be made by its manufacturer, is not guaranteed or endorsed by the publisher.

## Supplementary Material

Supplementary Video 1 Migrating myoclonus in patient 4 varied, and involved only one limb, or multiple limbs synchronously.

Supplementary Video 2 Electrographical artifacts and myoelectric activities were observed along with myoclonus in patient 4. Migrating myoclonus can involve only one limb, or multiple limbs synchronously, or can spread from one limb to other limbs.
